# Barriers to Recovery from Opioid Use Disorder Reported by Women During 2020: Insights for the Next Public Health Emergency

**DOI:** 10.3390/ijerph23030409

**Published:** 2026-03-23

**Authors:** Melissa K. Ward, Ayesha Jafry, Sarah Coleman, Sofia B. Fernandez, Tendai Gwanzura, Eric F. Wagner

**Affiliations:** 1 Department of Epidemiology, Robert Stempel College of Public Health and Social Work, Florida International University, 11200 S.W. 8th Street, AHC5-490, Miami, FL 33199, USA; 2 School of Social Work, Robert Stempel College of Public Health and Social Work, Florida International University, 11200 S.W. 8th Street, AHC5-536, Miami, FL 33199, USA; 3 Community-Based Research Institute, Florida International University, 11200 S.W. 8th Street, AHC5-536, Miami, FL 33199, USA

**Keywords:** opioid use disorder, women, barriers, recovery, COVID-19 pandemic, emergency preparedness

## Abstract

**Highlights:**

**Public health relevance—How does this work relate to a public health issue?**

- This research describes how access to treatment and recovery services for opioid use disorder was disrupted in 2020, as reported by women in recovery in the United States.
- By understanding these challenges, communities can better prepare to protect vulnerable populations from interruptions in treatment and support that can lead to recurrence of substance use problems and overdose during crisis situations.

**Public health significance—Why is this work of significance to public health?**

- The findings provide crucial insights for members of the recovery community, healthcare providers, treatment facilities, and decision makers.
- In the event of a future pandemic, these individuals should use a harm reduction approach to balance essential services for people with substance use disorders with important infection control measures.

**Public health implications—What are the key implications or messages for practitioners, policy makers and/or researchers in public health?**

- Harm reduction is critical for pandemic planning in substance use recovery settings.
- Lessons learned from 2020 can mitigate barriers to care and recovery in future public health emergencies.

**Abstract:**

This study seeks to inform emergency preparedness efforts by summarizing the pandemic’s impacts on access to opioid use disorder (OUD) recovery support as reported by women in recovery. In-depth interviews were completed with adult women in recovery from OUD. We used a primarily deductive approach to coding and analysis. Two coders analyzed transcripts; discrepancies were resolved through discussion. Seventeen women completed interviews from June to October 2020. Pandemic impacts primarily focused on engagement in care and retention at community and interpersonal levels. Community-level barriers to engagement included facilities’ halting intake of patients and fear of COVID-19 infection in treatment settings. Interpersonal barriers to engagement included loss of childcare support and the sudden transition to virtual services. Community-level retention barriers included perception of facility staff’s lack of adherence to infection prevention protocols and strict enforcement of infection prevention protocols on residents within facilities. Interpersonal barriers to retention included reduced availability of mutual aid meetings. Participants also highlighted how the pandemic worsened the addiction crisis and increased women’s caretaking burden. Leaders and administrators must be prepared to simultaneously balance responses for two public health crises: a novel infectious disease and addiction. Lessons learned from the pandemic can mitigate barriers to care and recovery when future emergencies arise.

## 1. Introduction

The onset of the pandemic in 2020 exacerbated another public health emergency in the United States: the opioid crisis. During the pandemic, drug overdose deaths increased more than what was predicted in its absence [1,2]. This increase has been associated with social isolation and insufficient care services which diminish an individual’s progress toward recovery and management of opioid use disorder (OUD) [3,4]. Additionally, access to medications for opioid use disorder (MOUDs) was disrupted [5,6].

Accessing OUD care for women has had long-standing barriers. These barriers have been documented in previous work and include fears related to child custody, insufficient childcare, stigma, discrimination related to the use of MOUD, lack of health insurance, and lack of adequate transportation [7,8,9]. The pandemic exacerbated these challenges. In Kansas, women recovering from substance use disorders reported that the pandemic negatively impacted their social relationships, which they identified as critical for their recovery [10]. In a study in North Carolina, before the pandemic, approximately half of responding clinics treating OUD reported accepting pregnant patients; during the pandemic, only 34% reported accepting pregnant patients [11].

Qualitative reports from people in recovery from OUD on the barriers to care and support they witnessed and experienced during the pandemic are critical to generating lessons learned for sustaining recovery support during future public health emergencies. The purpose of this study is to summarize the impacts of COVID-19 on access to care and recovery support for OUD as reported by women in recovery in the United States (U.S.) during the early months of the pandemic. By understanding participants’ experiences, decision makers and leaders can better prepare to protect vulnerable populations from treatment and recovery support disruptions during crisis situations in the future.

## 2. Materials and Methods

A larger qualitative study examining barriers to care for women with OUD was planned prior to the COVID-19 crisis. After it was evident the planned in-depth interviews would be conducted during the ongoing pandemic, a question specific to the impact of the pandemic was added to the semi-structured interview guide, following questions about the participant’s recovery experience and considerations women must take into account when seeking care for opioid use problems. The prompt was: “Now I’d like to ask you a question about current events. As you know, we are all currently experiencing the COVID-19 Pandemic. In your opinion, how do you think this may impact women seeking help for opioid use problems?” Here, we focus on the analysis of responses to this prompt.

Participants were women 18 years old or older who self-identified as being in recovery from OUD and resided in the U.S. Women were recruited using purposive (through recovery community organization networks) and snowball sampling (through word of mouth and referral from other participants) techniques. Confidential, in-depth, semi-structured interviews were conducted virtually through Zoom or phone, depending on participant preference and access. Interviews were transcribed verbatim. All participants provided electronic consent prior to the interview and all procedures were approved by the Florida International University Social and Behavioral Institutional Review Board.

We used a primarily deductive approach to codebook thematic analysis to examine responses to the prompt about the impact of the pandemic [12]. An initial codebook to analyze results was developed using two conceptual frameworks: the OUD Cascade of Care [13] and Levels of Influence as identified by the National Institute on Minority Health and Health Disparities (NIMHD) Research Framework [14]. Additional themes related to the impact of the pandemic were added to the codebook as they emerged from the interviews. Two coders (MW and SC) independently analyzed interview transcripts; any discrepancies were resolved through discussion and consensus. NVivo 15 (Lumivero, Denver, CO, USA) was used to manage and code data. The study followed the Standards for Reporting Qualitative Research [15].

## 3. Results

A total of 17 women in recovery completed in-depth interviews from June to October 2020; participant characteristics are shown in Table 1. Participants resided in three U.S. regions: the South (n = 7, 41.2%), the Northeast (n = 7, 41.2%), and the West (n = 3, 17.6%). Interviews concluded when data saturation related to the broader study was achieved. Study participants described a range of impacts on them personally, including restricted activities, increased appreciation for MOUD, loss of employment, and loss of household income. One participant shared an anecdote demonstrating the pandemic’s impact on employment of people in recovery who work as peers in substance use treatment facilities:

*…as COVID started to hit…they had to let… I want to say 20 to 30 people go. Just because numbers were tanking, which isn’t usual for the trends here… my supervisor was like ‘Of course we want you back in here.’… And they love hiring people who are in recovery, just because it’s super helpful for them… I at first was like ‘Okay, well, I’m going to get to go back. I’m going to get to go back.’ And then… we have this second wave of COVID hitting, I’m like ‘I have no idea when I would get to go back.’*

When describing the pandemic’s impact more broadly, participants’ comments primarily focused on two stages along the OUD Cascade of Care [13] at two Levels of Influence [14]: engagement in care and retention at community and interpersonal levels. A majority of comments focused on barriers to recovery support during the pandemic, although some participants also noted facilitators. Table 2 shows a summary of the themes that arose during interviews and demonstrative quotes. Additionally, participants noted the ways the pandemic worsened the addiction crisis, and the broader impacts of the pandemic on women.

### 3.1. Engagement in Care

At the community level, a major barrier to engagement in care discussed by participants was facilities’ halting intake of new patients. This was particularly concerning for women who needed admission to regain custody of their children. For example:

*…a lot of people aren’t accepting new clients, so there’s these women who are ready to get clean, who have lost their children. They’re ready to get them back. But these facilities aren’t accepting nobody because of the pandemic. I couldn’t imagine. I just really could not imagine.*

Participants also noted the difficulties facilities faced when enforcing infection control and prevention measures, and clients’ subsequent fear of COVID-19 infection that could impact vulnerable family members:

*…none of the clients in the treatment center did wear the mask... So I think that can be a fear for people, especially if they do have children or family members they live with, you’re contracting the virus and bringing it back home.*

Other barriers to engagement at the community level included hesitancy to ask for ways to interact with providers in ways that may reduce disease risk and the discontinuation of in-person tours (see Table 2 for sample quotes).

At the interpersonal level, participants noted that the sudden loss of childcare assistance from support systems women could previously rely on impacted their ability to access treatment. For example:

*…a lot of women, even single moms… can’t even just count on their parents to maybe watch them that night and they go to a 30-day program… they don’t really have that option at all. They need somebody that’s going to be there 24/7 with the kids.*

Participants emphasized the importance of interpersonal interactions for engagement in care and discussed how the sudden transition to virtual mutual aid groups posed difficulties in this respect. One participant noted that virtual meetings “can feel really out of place and weird for some people who are newly trying to get sober.” Another discussed how hard finding virtual meetings may be for those who are new to recovery or have limited access to cell phones: “Unless you have someone in the rooms that’s willing to walk you through that process or that’s willing to let you use their phone with them to check into a meeting, you’re screwed.” Others described challenges individuals experiencing homelessness may encounter when trying to access virtual meetings.

One facilitator to engagement in care noted at the interpersonal level was that stay-at-home orders might allow people requiring inpatient treatment to access it without explaining their absence to their acquaintances: “nobody will know because I’ve been quarantined for a few weeks anyway. I could just say I was quarantined…”

### 3.2. Retention

At the community level, one barrier to retention and potential loss of recovery was facility staff not adhering to infection prevention protocols when they were outside of the facility, resulting in exposure of clients and quarantine, causing in-person group meetings to be cancelled. Additionally, when this occurred, no alternatives to in-person meetings were offered (see Table 2 for example quotes).

Overly strict enforcement of pandemic rules was also mentioned as a barrier to retention. In the example below, a participant relayed an anecdote about the impact a playground closure had for a fellow resident in a supportive housing complex:

*…a mom left last Saturday because, they shut the playground down here and she wanted to take her son to the playground. So in the middle of the quarantine, she just had her son like hop the fence and played on the playground… of course the director drove out here and yelled at her about it. And she packed her stuff and left.*

A facilitator at the community level included facilities’ ability to navigate and adapt to pandemic restrictions and prevention measures in order to continue providing MOUD (Table 2).

At the interpersonal level, participants described the detrimental effects of the reduced availability of mutual aid meetings, which they deemed as critical to maintaining recovery. They noted the difficulty of navigating and enforcing infection prevention measures when they held meetings in their homes to address this gap. They also discussed the loss of community and support they felt from in-person interactions when meetings shifted to virtual formats. They felt the shift to virtual meetings reduced the quality of their interactions with or ability to provide support to their peers in recovery. One participant noted how the lack of in-person meetings made it harder for women to find other women in recovery, which is important for building networks of recovery support:

*…somebody told me once the women in this lifestyle are going to be the ones to save your life… It’s not like all these women go to the same Starbucks… now if they’re not having those meetings because places are closed [because] of COVID-19... It’s really, really sad.*

While participants highlighted the negative aspects of virtual mutual aid groups, they generally agreed that the widespread availability of virtual meetings was a good option to have, noting that the improved accessibility helped individuals who were homebound, elderly and residing in nursing homes, or individuals who were more introverted connect to peer support meetings. One participant shared how critical it was to her recovery during a period of quarantine when she did not have the ability or transportation to attend in-person AA meetings. This participant went on to describe how you can find a wide variety of virtual groups on the Intergroup AA website, hosted from locations around the world [16]. She had not heard of virtual meetings prior to the pandemic, and noted the importance of virtual meetings beyond the pandemic, particularly for mothers with childcare needs:

*…even some days… what, if it’s pouring rain one day or you don’t have a babysitter or something comes up, you can just plug in on your phone and get the spiritual fulfilling that you need for that day. So I really hope this stays on as long as I’m alive cause I know like I’m going to keep doing it.*

An additional facilitator to retention at the interpersonal level was having existing networks for recovery support prior to the pandemic (Table 2).

### 3.3. Additive Effects: The Pandemic and Addiction

Participants described the additive impact of the pandemic in relation to the addiction crisis:

*…we have this health crisis, but people are literally dying from untreated alcoholism and when we isolate addicts even further… we take away their 12 step meetings and we limit the treatment centers they can go to… It’s almost contributing to people not being able to get help.*

A participant succinctly summarized the impact of losing access to social networks: “it’s easier to die right now… isolation is very terrible for addicts.” Another participant noted that individuals with substance use disorders may underestimate the urgency of getting help: “…all nonessential appointments were canceled… if I was still using, I could easily just be like, ‘Oh, well, this isn’t essential. There’s a pandemic, I should just deal with this later.’” Participants also described how unemployment caused by the pandemic could exacerbate substance use by simultaneously creating more free time for substance use and by limiting the financial ability to enter treatment:

*…a lot of people are out of work, so you have a lot more time to acquire drugs and get high… a lot of places that are just not working right now, or if you’re… laid off, I think that that could definitely play a part in one, not having the money to seek treatment centers, and two, just having too much time on your hands.*

### 3.4. The Impact of the Pandemic on Women

In addition to exploring the ways the onset of the pandemic disrupted engagement and retention in care and support, participants also pointed out the negative impacts of the pandemic on women-led occupations, such as waitressing, and the challenges caused by the increased caretaking burden women experienced. This was summarized by one participant:

*I think that women are feeling the impact of COVID-19 more because… they carry a lot more than men… they carry an energetic weight of things… they’re probably working and they’re probably having to take care of their children at home. And they’re probably cooking and they’re probably cleaning and they’re probably doing everything that they did before, but like ten times more and also more stressed out because of everything that’s going on in the world…*

## 4. Discussion

We sought to examine the impacts of the early months of the pandemic on access to care and recovery support for OUD as reported by women in recovery. Participants provided first-hand accounts of the pandemic’s now well-documented impact on substance use and behavioral health services. It exacerbated existing barriers to care, particularly for individuals with OUD and other substance use disorders, who faced intensified challenges such as financial instability, social isolation, and heightened stress [17]. Healthcare providers and clients faced significant information gaps and inconsistencies in infection control guidelines, often due to conflicting messages from government agencies, the media, and other sources [18]. Behavioral health providers encountered further complications in implementing these measures due to the nature of their services, the vulnerable populations they interact with, and their often limited resources [18]. Structural limitations, such as the physical space needed for social distancing, staffing shortages, inadequate quantities of personal protective equipment (PPE), and limited access to rapid testing, further impeded service delivery [18,19]. Treatment centers reduced bed capacity by as much as 50%, creating financial difficulties for facilities reliant on bed-based reimbursements [19]. Additionally, restrictions on in-person support meetings and visits contributed to feelings of isolation, leading some clients to leave treatment earlier than recommended [19].

In response to these barriers, providers adopted multiple strategies to sustain service delivery while reducing the spread of disease. As described by Baloh and colleagues, standard infection control practices included symptom screening at entry, enforcing mask use, enhancing hand hygiene, and implementing social distancing measures [18]. Some facilities installed physical barriers, upgraded ventilation systems, and adjusted facility layouts to improve safety [18]. Residential treatment programs discontinued in-person visitations and adjusted housing arrangements to increase spacing between clients. Some required negative test results before admission, particularly as testing availability improved [18]. Many outpatient programs transitioned to telehealth services, using a mix of electronic record systems, Health Insurance Portability and Accountability Act-compliant teleconferencing software, and telephone-based services [18]. While in-person services were still necessary in some cases, such as for MOUD and drug screening, providers attempted to limit risks by incorporating telehealth when possible [18]. To ensure continued access to counseling and peer support services, many providers utilized telehealth for both individual and group sessions [20]. However, group services were often subject to additional restrictions, including limitations on the number of participants and specific telehealth requirements, such as the prioritization of video over audio-only communication [20].

Participants’ comments reflected both a fear of infection due to exposure from treatment and recovery facilities (along with fears of subsequently exposing vulnerable family members) and the dangers of social isolation for people with substance use disorders. For these women in recovery from OUD, social support was deemed a critical service; community was considered as important as medication for OUD. In practice, during the pandemic, healthcare administrators and providers treated social support as elective care. This is an important lesson that must be translated to pandemic preparedness, since the end of the Public Health Emergency Declaration on 11 May 2023 does not preclude us from experiencing another infectious disease outbreak or global pandemic in the future [21,22].

Harm reduction strategies for OUD continue to grow in popularity, and a harm reduction approach is critical for pandemic planning in substance use treatment and recovery settings as well [17]. If individuals with OUD require social interaction as part of their recovery, administrators can use lessons learned from the pandemic to plan for such interaction in the context of future infectious disease outbreaks [23]. For example, when interaction is needed, outdoor settings should be encouraged, and for indoor settings, strategies to improve ventilation and indoor air quality should be emphasized as necessary resources to maintain essential services [24,25]. High quality face masks should be made available, and health communication strategies should be used to highlight the importance of masks for keeping others safe, particularly in indoor spaces and for those who may be immunocompromised or have immunocompromised household members [24]. If vaccines become available, vaccination should also be promoted as a harm reduction strategy, although communication strategies must recognize that this community has great distrust of the pharmaceutical industry due to the role it played in the opioid crisis. In the context of a novel infectious disease, communication strategies should also highlight uncertainty and the potential for shifting guidelines as expert knowledge changes and improves [24].

Furthermore, to strengthen the resilience of substance use disorder treatment systems, providers should have greater access to PPE, sanitization supplies, and rapid testing capabilities [18]. Long-term planning should prioritize infrastructure modifications, including the expansion of housing for individuals in recovery, the creation of private spaces for telehealth services, and adjusting outdoor spaces for socially distanced meetings and visits [19]. Virtual recovery options can expand access for individuals who face ongoing barriers to in-person care, such as transportation, mobility, caregiving demands, or residence in rural areas with limited providers. Investments in digital infrastructure and digital literacy training will be crucial for sustaining telehealth accessibility, particularly for underserved populations. Policy adaptations that emerged during the pandemic, such as relaxed telehealth regulations and expanded insurance coverage, should be evaluated for long-term implementation to improve care accessibility and continuity.

Participants highlighted how difficult the rapid transition to virtual services was, particularly for individuals who were newly seeking care and support and for those who did not have access to devices required for virtual services. Again, using lessons learned, treatment and recovery settings should create plans to ease a sudden transition to virtual services and maintain equity in service availability in case it is required in the future. Participants’ discussion of how the pandemic increased women’s caretaking burden aligned with other reports documenting this phenomenon [26]. Strategies to address additional barriers to recovery women may face during a public health emergency, such as heightened caretaking responsibilities, should also be an essential component of emergency planning.

A major strength of this study is its focus on women’s lived experiences navigating OUD recovery during the early pandemic in 2020. Additionally, data were collected within the first year of the pandemic, when impacts were actively being experienced by participants. The qualitative descriptions offered here provide depth and detail unavailable through quantitative data. This study is also subject to several limitations. While the experiences provided by participants provide an important contribution to the literature on the challenges related to the provision of care and support for OUD during the pandemic, our small sample size limits generalizability to the general population or to more heterogenous groups. Furthermore, the parent study originally planned to explore barriers to care for women with OUD; since it was designed prior to the onset of the pandemic, the original research objective did not include the pandemic’s impact on participants. Additionally, since the in-depth interviews were conducted virtually due to pandemic restrictions, it is possible that the nature of the interactions with participants may have been improved if the interviews were conducted in person.

## 5. Conclusions

Participants commented on a number of areas related to engagement in care and retention that highlight the importance of emergency pandemic planning. Leaders and administrators must be prepared to balance responses for two potentially deadly public health crises: a novel infectious disease and addiction. This is a challenging balance to achieve, because the interventions for one may interfere with the interventions for the other. However, lessons learned from the pandemic can mitigate barriers to care and recovery when future emergencies arise.

## Figures and Tables

**Table 1:** Characteristics of women in recovery from opioid use disorder participating in in-depth interviews during the pandemic, June–October 2020.

Characteristics	N = 17
	n (%)
Age (mean, range)	28.88, 24–35
Race	
White	16 (94.1%)
Asian	1 (5.9%)
Ethnicity	
Hispanic/Latino	3 (17.6%)
Non-Hispanic/Non-Latino	14 (82.4%)
Region of Residence	
South	7 (41.2%)
Northeast	7 (41.2%)
West	3 (17.6%)
Number of Children Cared For	
None	9 (52.9%)
1	7 (41.2%)
2	1 (5.9%)
Marital Status	
Single	7 (41.2%)
Married/living together	1 (5.9%)
Widowed	1 (5.9%)
Separated/divorced	2 (11.8%)
Dating/In a relationship	6 (35.3%)
Annual Household Income	
Less than $30,000	8 (47.1%)
$30,000 to less than $50,000	3 (17.6%)
$50,000 to less than $75,000	3 (17.6%)
$75,000 to less than $100,000	
$100,000 or more	3 (17.6%)
Employment Status	
Employed	11 (64.7%)
Unemployed	6 (35.3%)
Highest Level of Education	
High school/GED	3 (17.6%)
Some college	6 (35.3%)
College graduate	5 (29.4%)
Vocational/trade school	3 (17.6%)
Health Insurance	
None	3 (17.6%)
Medicaid	7 (41.2%)
Employer-based	3 (17.6%)
Participant or family member buys on their own	1 (5.9%)
On parent’s insurance	2 (11.8%)
Insurance via Veteran’s Affairs	1 (5.9%)

**Table 2:** Sample quotes from women in recovery from opioid use disorder participating in in-depth interviews during the pandemic, June-October 2020.

OUD Cascade of Care	NIMHD Research Framework Level of Influence	Barrier/ Facilitator	Theme	Sample Quote
Engagement in Care & Support	Community	Barrier	Fear of COVID-19 infection	“Some people do not want to go to the treatment facility based on that. They’re scared. The idea of death scares people… If they will actually call the agencies that they’re going to and tell them ‘Hey I’m not really comfortable going, is there any kind of things that I could do? Can we meet outside? Can we [do] something else?’ They will get the assistance they need. It’s just, people need to be comfortable and ask… I think people don’t want to be a bother. I know I didn’t. I can’t answer for nobody else, but I think it’s just lack of knowledge, if people would just know everything first off instead of being nervous…”
Engagement in Care & Support	Community	Barrier	Facilities stopped intake	“…the place I’m at… we’ve got like three or four open apartments and they’re not taking anyone right now… I think that’s really scary… that could be preventing a lot of women that genuinely want to be here… that could be stopping them from getting their kids back… I think that’s the biggest thing is places not taking people right now.”
Engagement in Care & Support	Community	Barrier	Facilities transitioned to virtual tours	“…it was kind of like turning people off of trying to come get help because they’re like ‘Well, I want to see where I’m going to go.’ And then… having to adjust technology to kind of catch up with COVID was difficult for the treatment facility as a whole… we had to start limiting in person tours. And so we then had to try and figure out how to do like 3D tours… the hardest thing where it’s like ‘Okay, well we need to let these cameras in for this 3D capture of facility, but we also still have clients here that we can’t kick out today.’”
Engagement in Care & Support	Community	Barrier	Difficulty navigating infection prevention and control measures	“I know my treatment center when I was there, it was during pandemic and they were trying to enforce us to wear masks, but pretty much, no, none of the clients in the treatment center did wear the mask. Um, and you know, it was co-eds here with men and women from all over, um, new England, if not all over the country. So I think that can be a fear for people, especially if they do have children or family members, um, they live with you’re contracting the virus and bringing it back home.”
Engagement in Care & Support	Interpersonal	Barrier	Virtual mutual aid group meetings aren’t the same as in-person meetings	“...can feel really out of place and weird for some people who are newly trying to get sober.”
Engagement in Care & Support	Interpersonal	Barrier	Virtual mutual aid group meetings can be difficult to learn about and access	“…now it’s like, if you don’t have a computer, you don’t have a FaceTime or something like that, you’re pretty much screwed…half these times, these people in the street, they don’t have access to the internet the way that people that have been doing a little bit better in their life do.” “A lot of people either are homeless or they might not have access to a computer, or they might live in an environment that’s not safe and that they don’t feel comfortable being in.”
Engagement in Care & Support	Interpersonal	Barrier	Lack of childcare support that was present prior to the pandemic	“…a lot of women, even single moms… can’t even just count on their parents to maybe watch them that night and they go to a 30-day program… they don’t really have that option at all. They need somebody that’s going to be there 24/7 with the kids.”
Engagement in Care & Support	Interpersonal	Facilitator	Quarantine provides cover for going to inpatient treatment	“I could also see the aspect of like, well, nobody will know because I’ve been quarantined for a few weeks anyway. I could just say I was quarantined…”
Retention in Care	Community	Barrier	Overly strict enforcement of infection prevention and control measures	“…a mom left last Saturday because, they shut the playground down here and she wanted to take her son to the playground. So in the middle of the quarantine, she just had her son like hop the fence and played on the playground… of course the director drove out here and yelled at her about it. And she packed her stuff and left.”
Retention in Care	Community	Barrier	Facility staff not adhering to infection prevention protocols	“…up until about two weeks ago masks were not required here. Little crazy… we don’t go anywhere, but the problem is the staff does… the staff doesn’t live here, we do. And so when they leave, they’re around family, they’re around friends, they’re out at the store… and then we’re around them…. two staff members had the virus and came to work and exposed everyone. So we were on quarantine for two weeks and just got off yesterday… now masks are still not required, but they’re preferred… We haven’t had group for two weeks, but when we were having group, we were not really following any sort of 6 feet apart guidelines or masks.”
Retention in Care	Community	Barrier	Lack of alternatives to in-person meetings	“A lot of the moms here… came in straight from the street and, you know, had a couple of weeks clean. And then to be put on this, you know, two-week isolation. Like I can’t imagine that… I really feel like they should have brought around packets for everyone… something to keep them busy and you know focused on recovery…”
Retention in Care	Interpersonal	Barrier	Negative impact of stopping mutual aid meetings	“…those 12 step meetings are vital part of my recovery… being able to share those experiences with other people, it’s almost like you’re taking away my medication when you take away those meetings.”
Retention in Care	Interpersonal	Barrier	Lower quality interaction in virtual mutual aid meetings	“I’m actively looking for sponsors and it’s weird. It’s weird. I can’t just go into a meeting and be like, ‘Yo, you know, anybody?’ and like raising my hand… there’s not as much of an interpersonal connection.”
Retention in Care	Interpersonal	Barrier	Difficulty finding communities of women in recovery	“…somebody told me once the women in this lifestyle are going to be the ones to save your life… It’s not like all these women go to the same Starbucks. So how do you like know [how to] reach out, especially for somebody who’s new… there used to be a meeting list, but now if they’re not having those meetings because places are closed of COVID-19, it’s sad. It’s really, really sad.”
Retention in Care	Interpersonal	Barrier	Difficulty enforcing infection control and prevention measures at self-sponsored meetings	“I mean, we literally at our recovery homes, we had to start meetings at our house and, you know, we had 50, 60, 70 people here… we would require masks and things like that. And they would refuse and stuff like that, but we don’t want to be the bad guys who are not following the rules and not following the laws, like, because we teach people to follow the laws…”
Retention in Care	Community	Facilitator	Navigation around pandemic restrictions to continue providing MOUD	“…but I know that, so I have someone who works at a methadone clinic and she was saying that it’s open, and they’ve worked to navigate around COVID regulations to be able to, or they still follow COVID regulations to be able to, have their clientele be able to still come in and receive their methadone. And so, I’m not sure what they’re doing with group therapy I’m assuming over Zoom, I’m not sure, but they’re still doing these things…so I know that right now with COVID, they’re still allowing clientele into the clinic. Which is good.”
Retention in Care	Interpersonal	Facilitator	Virtual mutual aid groups facilitate access to recovery support	“I was on Facebook and someone sent me like a request to join a group called Zoom AA meetings… this was at the very beginning of the virus. So I joined the group… I was like really sad… I was clean, but I just wasn’t happy. And I just felt really low… I signed on to a meeting… and I just bawled my eyes out and just shared with them and there was like a hundred people in there and they were from all over… I found my sponsor in that meeting… she’s still my sponsor today…”
Retention in Care	Interpersonal	Facilitator	Having mutual aid communities prior to the pandemic facilitated support during the pandemic	“I know the rooms that have been together for a minute and the people that have been with them for a minute, through their contact and their networks, they’re getting together and they’re all face timing or whatnot… they’re still finding ways to get together.”

## Data Availability

The datasets presented in this article are not readily available due to privacy considerations. Requests to access the datasets should be directed to Melissa K. Ward.

## Abbreviations

The following abbreviations are used in this manuscript:

MOUD	Medication(s) for Opioid Use Disorder
NIMHD	National Institute on Minority Health and Health Disparities
OUD	Opioid Use Disorder
PPE	Personal Protective Equipment
